# Transplant Immunobiology: Many Answers Raising Even More Questions

**DOI:** 10.3390/ijms241612674

**Published:** 2023-08-11

**Authors:** Mihai Oltean

**Affiliations:** 1 The Transplant Institute, Sahlgrenska University Hospital, 413 45 Gothenburg, Sweden; mihai.oltean@surgery.gu.se; 2 Department of Surgery, Institute of Clinical Sciences, Sahlgrenska Academy at the University of Gothenburg, 413 45 Gothenburg, Sweden

Immunologic complications following organ, cell, or tissue transplantation still raise significant challenges related to their diagnosis and treatment. In spite of an increased understanding of transplant immunobiology and the availability of effective immunosuppression, acute rejection or graft-versus-host disease (GVHD) still occur in up to 50% of transplant recipients, depending on the graft type [1,2]. Increasing knowledge around its molecular mechanisms remains essential for further improving outcomes following organ and tissue transplantation. This Special Issue consists of four original research studies and one review, covering topics ranging from GVHD and xenotransplantation to biomarkers of intestinal rejection. All of these contributions have a distinct translational value and explore several relevant and pressing questions.

Graft-versus-host disease (GVHD) is a frequent and feared complication following hematopoietic stem cell transplantation (HSCT), and its development depends on multiple risk factors including donor–recipient matching and preconditioning regimens [2]. In their paper, included in this Special Issue, Tripathi et al. report the results of the molecular and functional characterization of 13 cytokine and cytokine receptor genes in 315 myeloablative, 10/10 HLA-matched donor–recipient pairs [3]. The authors found that HSCT recipients of grafts carrying the -1082GG interleukin-10 (IL-10) gene promoter region variant had a three-fold lower incidence of grade II–IV acute GVHD compared to IL-10 -1082AA graft recipients. IL-10 is a regulatory cytokine which inhibits the production of several pro-inflammatory cytokines such as IL-1β, IL-6, IFN-gamma, and tumor necrosis factor-alpha. In addition, IL-10 suppresses several cell types, including CD8+ T-cells and dendritic cell maturation, while stimulating the proliferation and differentiation of regulatory T-cells and Th2 cells [4]. Additionally, although high IL-10 levels are known to inhibit acute GVHD development, the current analysis differs from previous observations as earlier findings reported results from cohorts of HSCT patients receiving non-myeloablative protocols, while the circulating IL-10 levels were assessed only by ELISA. Interestingly, IL-10 -1082GG HSCT recipients did not experience an increased incidence of relapse, in spite of higher circulating IL-10 levels, while their overall survival was superior. The authors concluded that the incorporation of IL-10 variants into donor selection criteria, donor–recipient matching, and clinical management decisions may be useful and potentially able to improve patient outcomes following HSCT.

Xenotransplantation (transplantation across species) has long been envisioned as a practical solution to the organ shortage, yet significant immunological and ethical obstacles have hindered its clinical introduction. The logistic and ethical barriers related to the use of primates have prompted interest in the use of pigs, which are anatomically and physiologically similar to humans, as an economically viable and ethically accepted source of organs and tissues. Nonetheless, humans and pigs are incompatible at the level of several carbohydrate antigens on the cell surface, as humans possess natural antibodies against these antigens. Thus, the spectrum of hyperacute rejection has limited the use of porcine-derived grafts to decellularized tissues [5]. In spite of slow progress and early failures, the incremental knowledge gained in glycobiology and xenotransplantation as well as developments in gene editing technologies has opened the way for genetically modified animals by inserting or deleting specific genes, also recently paving the way for the transplantation of organs from genetically modified pigs to humans [6,7]. In this Special Issue, Yoon et al. provide further evidence that genetic engineering and the removal of carbohydrate antigens can overcome some of the incompatibilities generated by the xeno-context [8]. The subcutaneous implantation of meniscal tissue from minipigs lacking three different genes (alpha-1,3-galactosyltransferase (GGTA1), cytidine monophospho-N-acetylneuraminic acid hydroxylase (CMAH), and beta-1,4-N-Acetyl-Galactosaminyltransferase 2 (B4GALNT2)) into immunocompetent mice triggered milder infiltration of innate immune cells (mast cells, neutrophils, and eosinophils) compared with recipients of normal, unmanipulated meniscal tissue. Among its drawbacks, the study has a rather short follow-up period and a single sampling point after three weeks. Unfortunately, the authors did not analyze the adaptive immune response and lymphocyte infiltration, as non-vascularized tissue transplants tend to elicit a slower but effective immune response (i.e., tissue rejection) by about four weeks [9]. However, judging by the preserved morphology and the milder inflammatory infiltrate, it is likely that this approach successfully morphed the xenografts into allografts. Further long-term experiments on immunocompetent recipients, as well as biomechanical tests of the grafted tissue, would be needed to explore the long-term behavior of these valuable tissues.

Humoral immunity has long been recognized as one of the significant hurdles to solid organ transplantation. The antibody-mediated recognition of various antigens in transplanted organs has been associated with hyperacute or acute antibody-mediated rejection (AMR) and various strategies have been devised to tackle this obstacle. Complement-dependent lymphocytotoxic crossmatch, desensitization protocols through plasmapheresis, immunoabsorption, and B-cell depletion have allowed for an immunologically safer organ allocation or ABO-incompatible organ transplants, essentially abrogating hyperacute rejection and greatly reducing the occurrence of acute antibody-mediated rejection [10]. More recently, humoral immunity has been recognized as a key element in the development of chronic rejection [11]. As part of their paper included in this Special Issue, Reichert et al. used an elegant experimental model of chronic lung allograft dysfunction (CLAD) to provide further evidence that humoral immunity is activated in recipients of lung allografts during the development of CLAD [12]. Following rat single lung transplantation across a minor MHC class I mismatch, through a short course of cyclosporine and one intratracheal application of lipopolysaccharide to trigger mild, non-specific pulmonary inflammation, the authors found that intimal hyperplasia and perivascular and peribronchiolar remodeling, all typical features of CLAD, develop by day 40 after transplantation. B-cell infiltrates were seen in the perivascular and peribronchiolar regions, as well as in the alveolar space, while peribronchiolar/perivascular and intra-alveolar-Ig-positive cells increased in the allografts developing CLAD. Western blot analysis of the lung tissue confirmed increased content of both IgM and IgG. At the same time, allografts with CLAD displayed increased C4d immunoreactivity at the surface of respiratory epithelia and in the vasculature, corresponding to the external elastic lamina. Finally, in rats developing CLAD, the authors found autoreactive and alloreactive IgM and IgG antibodies. All of these changes seem specific to the organ that is developing CLAD as they were absent in isograft recipients and in the contralateral, native lung. While candidly admitting several potential methodological limitations, the authors conclude that all of these changes may be part of the pathogenesis of CLAD. This mechanistic study is important, and its findings are very relevant because it has a valid internal control in the non-transplanted contralateral lung and iterative analyses at several time points both in the tissue and in the serum, and it analyzes these changes in both allografts and isografts, thus taking into account any possible allo-independent factors.

Reactivity against leukocyte antigens (HLAs) has been long recognized as a central mechanism during allograft rejection. The pre-transplant crossmatch test between donor cells and recipient sera developed by Terasaki in the mid-1960s has allowed for better organ allocation and reduced immunologic risks after transplantation at a time when immunosuppressive medication was in its infancy. The initial classification of HLAs developed using early cytotoxicity assays has gradually been refined with the help of advancing technologies, and the extensive polymorphism of the original epitopes has now been solved by next-generation sequencing (NGS). These advances have also allowed for the definition of eplets, which are short sequences of amino acids which represent the HLA loci actually targeted by the HLA antibodies, with them being called “functional epitopes” as a result. Eplet-based matching has been suggested as a more precise strategy compared with HLA antigen matching [13].

In parallel, it has been repeatedly demonstrated that the occurrence of antibodies against donor HLAs after transplantation (the so-called de novo donor-specific antibodies, or dnDSAs), particularly those against HLA class II, is associated with the development of chronic allograft dysfunction [14]. A recent study by Lee et al. showed that HLA class II eplet mismatch was associated with dnDSAs, and combined analysis of eplet mismatch and tacrolimus levels may have prognostic value for the development of dnDSAs [15]. The mean number of HLA class II antigen mismatches was significantly higher in recipients who later developed HLA class II dnDSAs compared with recipients without dnDSAs. The authors went further to define risk groups according to the number of eplet mismatches and suggested several cut-offs grouped according to type. To this, their analysis also added tacrolimus intra-patient variability and found that insufficient exposure to tacrolimus is a significant risk factor leading to the development of dnDSAs. The authors concluded that eplet mismatch load can contribute to improved immunological risk stratification and may help in tailoring immunosuppression and personalizing post-transplant follow-up. As the relatively short follow-up duration in this study was only two years and the main event (biopsy-proven antibody-mediated rejection, ABMR) was relatively rare, the authors were not able to analyze any associations between eplet mismatch and the development of chronic ABMR.

The transplanted intestine is constantly exposed to external antigens, and its complex and dynamic microbiome makes intestinal graft immunology unique among transplantable organs. Intestinal grafts elicit a strong immunologic response due to their abundance in epithelial cells and tissue-resident immune cell populations. These circumstances have led numerous investigators to assume that the mechanisms of rejection in the intestine differ from those of other solid organs. In addition, in our review included this Special Issue, Professor Martin Rumbo and I summarized the experimental and clinical evidence of the roles of the direct pathway, the semi-direct pathway, and the indirect pathway of allorecognition in intestinal transplantation [16]. Our review also covers several emerging paradigms such as the two-way allorecognition model, the role of enterocytes as non-conventional antigen-presenting cells, and the importance of resident memory T cells in intestinal graft immunobiology. These peculiarities likely have direct clinical consequences and may explain the insufficiency of some immunosuppressive protocols, otherwise adequate for other solid organ transplants, to control the development of acute rejection. The review also recapitulates the attempts made to identify a non-invasive rejection marker, as intestinal grafts still lack a reliable test able to diagnose acute rejection with sufficient sensitivity and specificity. One conclusion is that virtually all candidate markers tested are directly “imported” from the field of inflammatory bowel disease, which may explain its low specificity in differentiating acute rejection from any other type of intestinal inflammation, be it infectious or immune. On the other hand, the review highlights that the same technological advances that permeate this Special Issue, namely the emergence of the “omics” and the abundant novel information thereof, have been used increasingly in the search for biomarkers [17,18]. These recent studies may open new avenues of research and ultimately identify a specific marker, either in the blood or in the stomal effluent/feces, that could ultimately replace the need for frequent graft endoscopies and mucosal biopsies, which currently represent the mainstay of graft surveillance.

## Abbreviations

ABMR—antibody-mediated rejection; CLAD—chronic lung allograft dysfunction; DnDSA—de novo donor-specific antibody; DSA—donor-specific antibodies; GVHD—graft-versus-host disease; HLA—human leukocyte antigen; HSCT—hematopoietic stem cell transplantation; Ig—immunoglobulin; IFN—interferon; IL—interleukin.
